# Effects of Le Fort I Osteotomy on Maxillary Anterior Teeth: A 5-Year Follow Up of 42 Cases

**Published:** 2010-01-08

**Authors:** Abolhasan Mesgarzadeh, Mohammad Hosein Kalantar Motamedi, Hengameh Akhavan, Tara Sarvghad Tousi, Peyman Mehrvarzfar, Pooyan Sadr Eshkevari

**Affiliations:** ^a^Tehran University of Medical Sciences, Tehran, Iran; ^b^Trauma Research Center, Baqiyatallah University of Medical Sciences, Tehran, Iran, and Azad University of Medical Sciences, Tehran, Iran; ^c^Azad University, Tehran, Iran; ^d^Drs Tousi and Eshkevari are in private practice dentistry, Tehran, Iran

## Abstract

**Aim:** The aim of this study was to assess the vitality of maxillary anterior teeth following Le Fort I osteotomy. **Materials and Methods:** A total of 245 maxillary anterior teeth in 42 patients were examined by several pulp vitality tests before surgery and 1 to 5 years postoperatively. Data were recorded in SPSS and were statistically analyzed by using Pearson, χ^2^, and Fisher exact tests. **Results:** This study showed a significant number (91%) of the maxillary anterior teeth to be sensitive to cold, 88.8% to electrical pulp test, and 89.4% to heat tests 12 months to 5 years following Le Fort I osteotomy. A total of 8 teeth (3.2%) had undergone root canal therapy (RCT) because they were nonvital and had developed apical lesions. Pain on percussion was observed in 5.7% of the teeth. External resorption was significantly associated with insensitivity (*P* < .05). Orthodontic therapy adds to this especially if excessive force is applied. **Conclusion:** A significant number of teeth had sensitivity after Le Fort I osteotomy. Only 3.2% needed RCT. When all vitality tests were negative, we used periapical radiolucency as the main criterion for judging pulp necrosis requiring RCT. It should be stressed that the outcomes of a single test cannot be considered as a reliable indicator for the presence or absence of pulpal or periapical disease or for RCT. Although the complications following Le Fort I osteotomy are few, follow-up is mandatory.

Orthognathic surgery may pose potential risks to the vitality of teeth. It may be the cause for the development of pulpitis or root resorption^1^^-^4 as well as pulp injuries,^5^ periodontal problems,^3^,^5^,^6^ and root damage.^5^,^7^ Post–Le Fort I osteotomy studies on humans and animals have led to controversial outcomes.^1^ Some reported that 29% of anterior teeth did not respond to electrical pulp test (EPT) following a 14-month follow-up period. In contrast, Kahnberg and Engström reported that 90% to 100% of teeth had given positive responses to EPT.^3^,^4^ Justus et al,^5^ using laser Doppler flowmetry following Le Fort I osteotomy, described an increase in pulpal blood flow between the first and third postoperative weeks. Patients should be informed of these issues that may occur following surgery.^4^,^5^ In most cases, however, follow-up appointments do not receive enough attention and a lack of knowledge regarding the probable pulpal sequelae is prevalent among patients who have undergone Le Fort I osteotomy. We aimed to assess the changes and vitality status of maxillary anterior teeth before and after standard Le Fort I osteotomy in patients referred to us from 2001 to 2005.

## MATERIALS AND METHOD

This study included 42 patients (32 women and 10 men) who were 18 to 50 years old (245 teeth) and who were treated by Le Fort I osteotomy; in all cases, the anterior bone-cut line was started from the piriform aperture 4 mm above the nasal floor (no possible association with the incisors) and 5 mm above the apex of the canine tooth. All patients had normal unrestored vital incisors preoperatively. Postoperatively, the maxillary anterior teeth were examined using heat, percussion, palpation, cold, and EPT, respectively. Mandibular anterior teeth were considered as control teeth for each patient. Each test was repeated at least 3 times for case and control teeth with 30-second, 1-minute, and 2-minute intervals, respectively. Responses in case and control teeth were assessed taking into account the following:

1. Discoloration was detected in a dry condition, under the illumination of the dental unit light (not necessarily pulp necrosis).
2. Periodontal pocket depth was measured in mesial and distal gingival sulci and recorded if it exceeded 2 mm (possible periodontal involvement).
3. A dry, rotating rubber prophy cup delicately pushed on the isolated, dry tooth surface provided frictional heat test (sensation positive in vital teeth). However, heat could cause pain in necrotized pulp, which was considered as a false-positive response. Instead, a pain sensation, which disappeared by the withdrawal of stimulus, reported by the patient due to the test was considered as true-positive response.
4. For the cold test, a sharp nonlingering pain response was considered as an indicator for vital (not necessarily normal) pulp.
5. Percussion test was performed by tapping a mirror end on the incisal edge of anterior teeth, with the mirror handle held perpendicular to the crown to detect not only the probable sensitivity but also the probable ankylosis (a solid sound indicative of loss of periodontal ligament around the tooth and probable ankylosis).
6. Palpation test was performed by fingertip pressure. Mild pressure was applied initially, avoiding probable severe pain.
7. Mobility was measured by putting the first finger on the lingual and the mirror's handle on labial surfaces. Orthodontic therapy adds to tooth mobility especially if excessive force is applied.
8. Cold test was performed by applying ethyl chloride spray (Vocko, Coltene, Del) on the isolated facial middle-third of each incisor's clinical crown. A short sharp response was not considered as an indicator because it might occur regardless of pulp status (normal or reversible or irreversible pulpitis). However, an intense and prolonged response was considered as an indicator for irreversible pulpitis. In contrast, necrotic pulps would not respond. Also, there was the possibility of obtaining a false-negative response in teeth with constricted canals (calcific metamorphosis).
9. Electric pulp test was performed using the Parkell device (Parkell Inc, Farmingdale, NY). In the case of obtaining negative response on the facial middle-third, each test was repeated on the palatal middle-third of the clinical crown.
10. Three postoperative periapical radiographs were obtained for each patient and were compared with the preoperative images. External resorption in outer root surface and surrounding bone was sought and recorded if present. Orthodontic therapy adds to this especially if excessive force is applied.
11. When all vitality tests were negative, we used periapical radiolucency as the main criterion for judging pulp necrosis requiring RCT.
12. Obtained data were classified by SPSS software and statistically analyzed using Pearson, χ^2^, and Fisher exact tests.

## RESULTS

**Figure 1:**
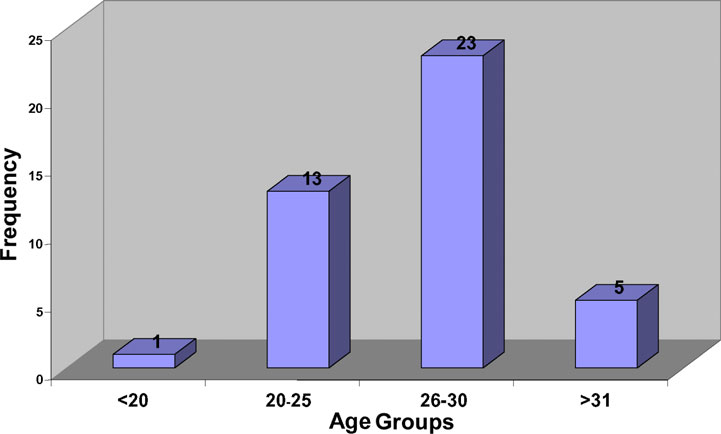
Age distribution of the patients in this study. It is of note that most patients undergoing surgery were in the 2nd to 3rd decades of life.

In this study, maxillary anterior teeth of 42 patients (245 teeth) were examined before and 1 to 5 years following Le Fort I osteotomy. Age distribution of the patients is presented in Figure 1. This study showed the majority (91%) of the maxillary anterior teeth to be sensitive to cold, 88.8% to EPT, and 89.4% to heat tests 12 months to 5 years following Le Fort I osteotomy. A history of orthodontic intervention before or after treatment was observed in 32 patients (76.2%). Eight (3.2%) of 245 anterior teeth required RCT following operation because of periradicular radiolucency and negative responses to cold, heat, and EPTs. Tables 1–3 show the results of EPT, percussion, thermal, and palpation tests and external resorption of maxillary anterior teeth following Le Fort I osteotomy 1 to 5 years postoperatively. A significant relation was observed between external resorption and the results of vitality tests (*P* < .05).

## DISCUSSION

Studies on revascularization by Bell et al^8^ emphasize the role of attached soft tissue in providing blood supply for the maxillary segment following Le Fort I osteotomy. If Le Fort I osteotomy is performed correctly, there is usually no risk of injury to the blood supply of the maxilla or dentition. However, transient reversible vascular ischemia of the pulp and direct injury of tooth apices serve as 2 major potential factors resulting in pathologic changes within the pulp.^9^,^10^ Also, on the basis of the studies conducted by Bell et al,^8^ maxillary osteotomy does not necessarily cause pulpal injury. This injury rather occurs when pulpal blood flow is severed. Thus, the bone cut is best executed far from the root apices (at least 5 mm). This study showed the majority (91%) of the maxillary anterior teeth to be sensitive to cold, 88.8% to EPT, and 89.4% to heat tests during follow-up (12 months to 5 years) after Le Fort I osteotomy. Pain on percussion, was observed in 5.7% of the cases.

In 1989, Vedtofte and Nattestad^3^ reported 6% of 617 maxillary teeth, mostly canines, to show a negative response to EPT. This was suggested to be because of the proximity of canines' long roots to the osteotomy site and is supported by our findings in which canines demonstrated a lower sensitivity (more negative responses) to EPTs.

In 1987, Kahnberg and Engström^4^ performed EPTs on 44 patients following Le Fort I osteotomy and observed a positive response in 100% of the teeth in an 18-month follow-up. In 1999, Mordenfeld and Andersson^1^ encountered a positive response to EPT in 89% of central incisors in a 12-month follow-up of 20 patients, following midline osteotomy. Similar to other studies, canines demonstrated less sensitivity to different tests probably because of their longer roots. This study supports the findings of other studies in the literature for most cases.

The assessment of the complications following Le Fort I osteotomy in the present study revealed external resorption in 30.6% of maxillary anterior teeth. In addition to the above-mentioned findings, we noticed a 5.3% tooth discoloration rate following osteotomy, but no statistically significant relationship was detected between pulp vitality and the discoloration. Buckley et al^11^ claimed in 1999 that Le Fort I osteotomy impairs the maxillary blood supply and results in noticeably decreased viability and discoloration in anterior teeth. Studies on humans and animals have shown the possibility of long-term ischemia in displaced maxillary segments. This temporary ischemia is responsible for the development of periodontal defects, pulpal degenerative changes, lack of an integrated bony unit, and loss of the whole displaced segment in some instances. Despite normal bone healing and normal tooth coloration following this surgical approach, important questions regarding its complications have been raised.^6^ It is recommended that patients refer for follow-ups and be informed of possible alterations in tooth sensitivity and discoloration. Also, the clinician should not rely on pulp vitality responses alone even after 1 year postoperation. It should be stressed that the outcomes of a single test cannot be considered as a reliable indicator for the presence or absence of pulpal or periapical disease or for RCT. We used the presence of periapical radiolucency as the main criterion for judging pulp necrosis requiring RCT when other vitality test results were also negative.

## CONCLUSION

Although a significant number of teeth had sensitivity after Le Fort I osteotomy, only 3.2% needed RCT. The assessment of the complications following Le Fort I osteotomy in the present study revealed external resorption in 30.6% of maxillary anterior teeth. A significant relation was observed between external resorption and the results of vitality tests (*P* < .05).

## Figures and Tables

**Table 1:** Maxillary central incisors test responses*

	Right (*n* = 42)		Left (*n* = 42)		Maxillary centrals (*n* = 84	
	Positive	Negative	Positive	Negative	Positive	Negative
Cold	38 (90.5)	4 (9.5)	39 (92.9)	3 (7.1)	77 (91.7)	7 (8.3)
Heat	39 (92.9)	3 (7.1)	39 (92.9)	3 (7.1)	78 (92.9)	6 (7.1)
EPT	39 (92.9)	3 (7.1)	39 (92.9)	3 (7.1)	78 (92.9)	6 (7.1)
Percussion	3 (7.1)	39 (92.9)	3 (7.1)	39 (92.9)	6 (7.1)	78 (92.9)
External resorption	17 (40.5)	25 (59.5)	16 (38.1)	26 (61.9)	33 (39.3)	51 (60.7)

*Values are expressed as number (percentage). EPT indicates electrical pulp test.

**Table 2:** Maxillary lateral incisors test responses*

	Right (*n* = 39)		Left (*n* = 39)		Maxillary laterals (*n* = 78)	
	Positive	Negative	Positive	Negative	Positive	Negative
Cold	37 (94.9)	2 (5.1)	39 (100)	…	76 (97.4)	2 (2.6)
Heat	37 (94.9)	2 (5.1)	35 (89.7)	4 (10.3)	72 (92.3)	6 (7.7)
EPT	34 (87.2)	5 (12.8)	37 (94.9)	2 (5.1)	71 (91)	7 (9)
Percussion	2 (5.1)	37 (94.9)	3 (7.7)	36 (92.3)	5 (6.4)	73 (93.6)
External resorption	15 (38.5)	24 (61.5)	13 (33.3)	26 (66.7)	28 (35.8)	50 (64.2)

*Values are expressed as number (percentage). EPT indicates electrical pulp test.

**Table 3:** Maxillary canines test responses*

	Right (*n* = 41)		Left (*n* = 42)		Maxillary laterals (*n* = 83)	
	Positive	Negative	Positive	Negative	Positive	Negative
Cold	33 (80.5)	8 (19.5)	37 (88.1)	5 (11.9)	70 (84.3)	13 (15.7)
Heat	31 (75.6)	10 (23.4)	37 (88.1)	5 (11.9)	68 (81.9)	15 (18.1)
EPT	31 (75.6)	10 (23.4)	35 (83.3)	7 (16.7)	66 (79.5)	17 (20.5)
Percussion	1 (2.4)	40 (97.6)	2 (4.8)	40 (95.2)	3 (3.7)	80 (96.3)
External resorption	7 (17.1)	34 (82.9)	7 (16.7)	35 (83.3)	14 (16.8)	69 (83.2)

*Values are expressed as number (percentage). EPT indicates electrical pulp test.
